# Persistent anterior tunica vasculosa lentis in multisystemic smooth muscle dysfunction syndrome: A case report

**DOI:** 10.1097/MD.0000000000026094

**Published:** 2021-06-04

**Authors:** Kaiqin She, Licong Liang, Fang Lu

**Affiliations:** Department of Ophthalmology, West China Hospital, Sichuan University, Chengdu, Sichuan Province, China.

**Keywords:** case report, congenital mydriasis, multisystemic smooth muscle dysfunction syndrome, persistent anterior tunica vascluosa lentis, retinal arteriolar tortuosity

## Abstract

**Rationale:**

Multisystemic smooth muscle dysfunction syndrome (MSMDS) is a genetic disease that affects multiple organs. The report here concerns a patient with MSMDS, who is known so far as the youngest among all the reported patients. In addition to the typical manifestations, we observed previously unreported ocular abnormalities, including persistent anterior tunica vasculosa lentis (TVL) and early-onset retinal arteriolar tortuosity, by the fluorescein angiography (FA).

**Patient concerns:**

The patient was admitted to the neonatal intensive care unit immediately after birth for a diagnosis of urinary system dysplasia during fetal life. After a thorough examination, the patient was found with patent ductus arteriosus, pulmonary hypertension, cerebrovascular disease, hypotonic bladder, intestinal malrotation, and congenital mydriasis. The FA of the eyes undertaken in her 6-week demonstrated perfused vasculature in the persistent anterior TVL and prominent retinal arteriolar tortuosity. The whole exome sequencing revealed a de novo heterozygous *ACTA2* gene missense mutation p.R179H.

**Diagnoses:**

The patient was diagnosed with MSMDS.

**Interventions:**

Follow-up observation.

**Outcomes:**

At the 3-month follow-up, no change of the ocular disease was observed.

**Lessons:**

The persistent anterior TVL in this case implies that *ACTA2* p.R179H mutation affects not only the smooth muscle cells but also the pericytes, and further affects the TVL regression. The prominent retinal arteriolar tortuosity in this 6-week-old infant indicates that the retinal arteriolar tortuosity can present early in MSMDS.

## 1 Introduction

Multisystemic smooth muscle dysfunction syndrome (MSMDS) is a rare genetic disease characterized by the dysfunction of smooth muscle cells (SMCs) throughout the body. It is caused by the heterozygous mutation of *ACTA2* gene. *ACTA2* gene encodes α-actin protein, which is the most abundant isoform in SMCs. SMCs are found in the wall of arteries and hollow visceral organs, lacrimal ducts, hair follicles and the iris. MSMDS, mostly caused by heterozygous missense mutation of the *ACTA2* altering arginine 179, represents the most severe *ACTA2* mutation-associated disease because of the early onset and highly penetrant vascular disease.^[1]^ It leads to aortic and cerebrovascular disease, pulmonary hypertension, hypotonic bladder, intestinal hypoperistalsis and malrotation, and ocular abnormalities.^[1]^

The most common ocular manifestation of MSMDS is congenital mydriasis. Late-onset and progressive retinal arteriolar tortuosity, and persistent pupillary membrane have been reported in some cases.^[2–4]^ Hereafter we will describe a patient with typical features of MSMDS and with the most severe ocular abnormalities, including congenital mydriasis, early-onset retinal arteriolar tortuosity and a previously unreported ocular manifestation, persistent anterior tunica vascluosa lentis (TVL).

## 2 Case report

This study was approved by the ethics committee of West China Hospital, Sichuan University. Written informed consent was obtained from the patient's parents. A female full-term born baby with a normal birth weight was admitted to the neonatal intensive care unit immediately after birth for a diagnosis of urinary system dysplasia during fetal life. Echocardiogram revealed patent ductus arteriosus, atrial septal defect, patent foramen ovale, tricuspid regurgitation, dilation of pulmonary artery, and pulmonary hypertension. Color ultrasound of abdominal demonstrated enlarged bladder, bilateral hydronephrosis, and ureter dilation. Abdominal CT scan revealed intestinal malrotation. Cranial magnetic resonance imaging was normal except for widening extracerebral spaces of the temporal lobes and left parietal lobe, and widening lateral fissures. Cranial magnetic resonance angiography demonstrated the absence of A1 segment of right anterior cerebral artery and abnormally straight course of the intracranial arteries. Ligation of patent ductus arteriosus was performed 2 weeks after birth.

**Figure 1:**
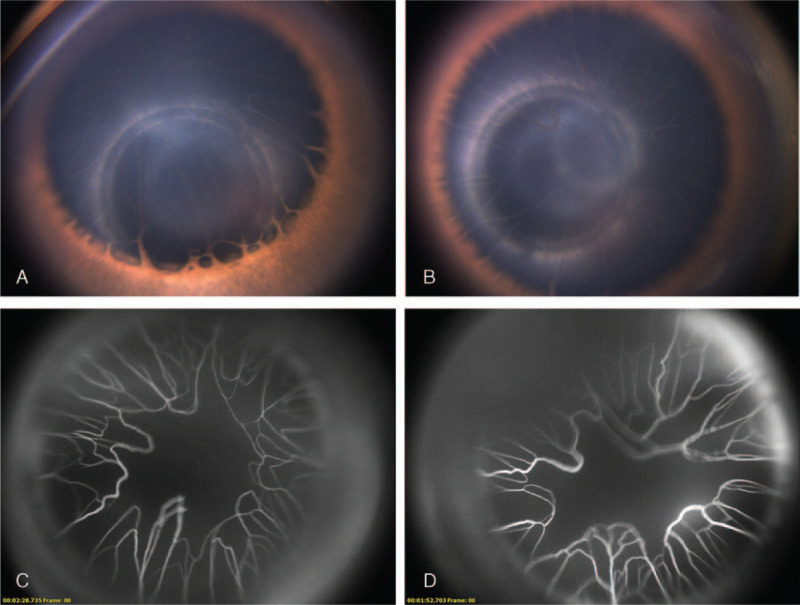
Photograph and fluorescein angiography of the anterior segment of both eyes. Photograph of the anterior segment of the right eye (A) and the left eye (B) showed the persistent anterior tunica vascluosa lentis (TVL), and the absence of iris crypts and furrows (A). Fluorescein angiography of the anterior segment of the right eye (C) and the left eye (D) showed the perfused vessels of TVL covering the anterior 2/3 of the lens surface with central sparing.

**Figure 2:**
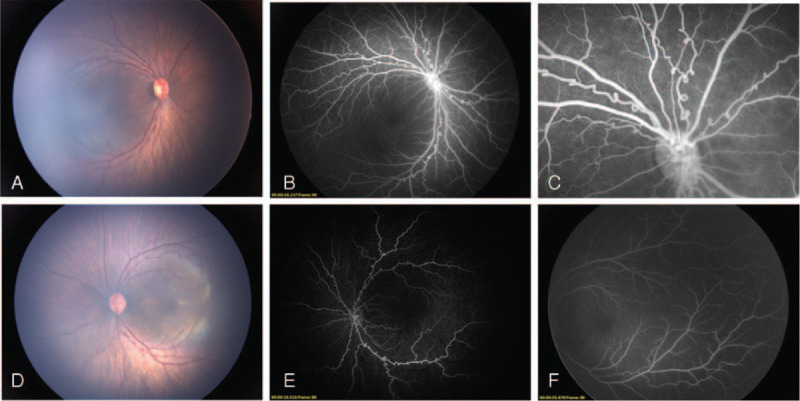
Fundus photograph and fluorescein angiography of both eyes. Fundus photograph of the right eye (A) and the left eye (D) showed the tortuosity of the retinal arterioles. Fundus fluorescein angiography of both eyes showed the distinct loops of the retinal arterioles (B and E) and the closer it became to the optic disc, the more frequently and severely the loops appeared (C). There was no leakage, venous or periphery vascular abnormalities (B and F).

An ophthalmic examination under anesthesia was conducted at the 6th week after her birth. It demonstrated fixed dilated pupils (5 mm) bilaterally, which were unresponsive to light and cycloplegic eye drops (Fig. 1A and B). The pupillary zone of the iris, from the pupillary margin to collarette, was narrow, while the rest of the iris was hypoplastic with the absence of crypts and furrows (Fig. 1A). The most exceptional finding was the persistent anterior TVL with vascular perfusion shown on fluorescein angiography (FA) covering the anterior 2/3 of the lens surface with central sparing (Fig. 1C and D). The retinal arterioles in both eyes were tortuous with multiple loops formation, which were more prominent in the vicinity of optic discs (Fig. 2A-E). No leakage, venous or peripheral vascular abnormalities was found on FA (Fig. 2B and F). Both eyes were affected symmetrically. No abnormality was found in her parents’ eyes. Whole exome sequencing revealed a de novo heterozygous *ACTA2* gene missense mutation p.R179H, confirming the MSMDS diagnosis along with the typical clinical manifestations. Since there is no evidence of active disease in the anterior nor the posterior of the eyes, we decided to observe and follow up the patient monthly for the first 3 months. At the latest 3-month follow-up, no change of the ocular disease was observed. The patient was asked to visit the ophthalmic outpatient clinic every 3 months during the next year.

## 3 Discussion

To the best of our knowledge, this is the first report that describes both the anterior and the posterior ocular features of MSMDS by FA demonstrating perfused vasculature in the persistent anterior TVL and prominent retinal arteriolar tortuosity. Meanwhile this 6-week-old girl is the youngest MSMDS patient among all the reported cases so far.

Persistent anterior TVL described here for the first time is considered to be a more severe condition of persistent pupillary membrane in previously reported cases, both of which are considered as incomplete regression of anterior TVL.^[2–4]^ The TVL, part of the primary hyaloid vasculature system, provides oxygen and nutrients to the lens from about 6 weeks gestation and regresses completely by the eighth month of gestation in normal eye development.^[5,6]^ Persistent TVL has been described in premature infants, aniridia, and congenital myotonic dystrophy.^[7–9]^ In both our case and the previously reported MSMDS cases with persistent pupillary membrane, the patients were born at full term. Pericytes are the major components of TVL walls and express α-actin in the large branches of TVL.^[10]^ We speculate that *ACTA2* p.R179H mutation affects not only the SMCs but also the pericytes, and further affects the TVL regression.

According to the fundus examination and FA, the retinal arteriolar tortuosity was the only retinal vascular abnormality in this case. In the previous reports, retinal arteriolar tortuosity was considered to be late-onset and progressive.^[2]^ Nevertheless, the prominent retinal arteriolar tortuosity in this 6-week-old infant indicates that retinal arteriolar tortuosity can present early. According to the FA, it is clear that the tortuosity is much more prominent in arterioles near the optic disc and even loops are formed. We speculate that it might result from the uneven distribution of α-actin in retina arterioles. The largest arterioles, near the optic disc, are richer in α-actin than arterioles after several branchings.^[11]^ The more α-actin arterioles contain, the more tortuous arterioles appear.

Congenital mydriasis, which is common in MSMDS, is due to the reduced contractility of iris sphincter and dilator muscles, resulting in fixed dilated pupils.^[4]^ The absence of iris crypts and furrows, which can be seen in congenital microcoria, may be the result of the dilator muscle hypoplasia.^[12]^ In summary, we observed congenital mydriasis, persistent anterior TVL and early-onset retinal arteriolar tortuosity in the eyes of a 6-week-old girl with a de novo heterozygous *ACTA2* gene missense mutation p.R179H causing MSMDS.

## Author contributions

**Conceptualization:** Fang Lu.

**Data curation:** Licong Liang.

**Investigation:** Licong Liang.

**Supervision:** Fang Lu.

**Writing – original draft:** Kaiqin She.

**Writing – review & editing:** Kaiqin She, Fang Lu.
